# Comparative analysis of pregnancy outcomes after emergency and elective cerclage: A systematic review and meta-analysis

**DOI:** 10.12669/pjms.41.9.12685

**Published:** 2025-09

**Authors:** Yiyi Zhang, Tianzhen Tang

**Affiliations:** 1 Yiyi Zhang Department of Obstetrics, Huzhou Maternity & Child Health Care Hospital, Huzhou, Zhejiang Province 313000, P.R. China; 2 Tianzhen Tang Department of Obstetrics, Huzhou Maternity & Child Health Care Hospital, Huzhou, Zhejiang Province 313000, P.R. China

**Keywords:** Cervical Cerclage, Meta-Analysis, Pregnancy, Ultrasonography

## Abstract

Cervical insufficiency poses significant risks to ongoing pregnancies, often necessitating interventions such as cervical cerclage. This review compares pregnancy outcomes in women who underwent emergency or elective cerclage. A comprehensive literature search of PubMed, Scopus, Embase, Cochrane library and Web of Science databases was conducted from the inception of these databases up to 30 April 2024. The risk of bias was assessed using the Newcastle Ottawa scale (NOS). Odds ratios (OR) and weighted mean differences (WMD) were calculated depending on the type of outcome using random-effects model. Overall, 27 studies were included. Gestational age of women who underwent emergency cerclage was significantly higher at cerclage (WMD of 6.500 and 5.698 weeks for physical examination-guided and ultrasound-guided cerclage, respectively, compared to elective cerclage). Emergency cerclage placement was associated with lower neonatal birth weights, particularly in the physical examination-guided group (WMD = -780.52 grams). The risk of PROM was higher in the physical examination-guided emergency cerclage group (OR = 2.197). However, the incidence of PROM was comparable in the elective and ultrasound-guided groups. Physical examination-guided cerclage was associated with significantly higher vaginal delivery rates compared to the emergency cerclage (OR = 1.750). Emergency cerclage, especially when guided by physical examination, is associated with later gestation at application and higher risks of adverse neonatal outcomes compared to elective cerclage.

**Registration:**

PROSPERO: CRD42024546566

## INTRODUCTION

Cervical insufficiency is a significant obstetric complication characterized by the inability of the cervical tissue to maintain a pregnancy in the absence of clinical contractions or labor.^1^ This condition is often diagnosed retrospectively after a history of painless cervical dilation leading to recurrent mid-trimester losses.^1^

Cervical cerclage, a surgical procedure that involves suturing the cervix closed to reinforce its strength, is commonly used to prevent pregnancy loss and preterm births in women with cervical insufficiency.^2^ The decision to place a cerclage is typically influenced by obstetric history and the timing of diagnosis. Based on that, the procedure may be classified as elective or emergency cerclage. Elective cerclage is often based on obstetric history and is usually performed in the second trimester for women with a history of cervical insufficiency,^3^ while emergency cerclage is considered in cases of visible cervical dilation in the second trimester that are not accompanied by the signs of labour or infection.^4^

The distinction between elective and emergency cerclage also reflects a difference in the underlying risk profiles and prognosis. Elective cerclage is typically planned and performed under less urgent circumstances, where the integrity of cervix is not yet severely compromised.^5^ Conversely, emergency cerclage is a reactive measure, often undertaken in a setting where the risk of pregnancy loss is imminent.^5^ Due to these differences, the outcomes of pregnancies following these procedures can vary significantly. Therefore, a careful evaluation of the relative effectiveness and safety of both approaches is crucial. However, the decision-making in cases of cervical insufficiency may be influenced by factors such as the degree of cervical change at the time of diagnosis and the presence of contraindications such as intra-amniotic infection.^6^ Furthermore, there is still no consensus on the timing and indications for placing a cerclage since clinical guidelines are primarily based on retrospective data and expert opinion rather than high-level evidence. While systematic reviews have evaluated specific cerclage indications such as physical examination-indicated cerclage and history-indicated (prophylactic) cerclage and recent meta-analyses have focused on the efficacy of emergency cerclage in singleton and twin pregnancies,^3^ no prior work has directly compared emergency versus elective cerclage outcomes within a single quantitative synthesis. Our study fills this gap by pooling and contrasting key perinatal outcomes across both strategies to inform optimal timing of cerclage placement.

This review aimed to compare the impact of emergency and elective cerclage on pregnancy outcomes to clarify the effectiveness and safety of both approaches.

## METHODOLOGY

### Literature search:

A comprehensive search of PubMed, EMBASE, Cochrane Library, Web of Science and Scopus was done from the inception of these databases up to 30 April 2024. The keywords and MeSH terms specifically related to “cervical cerclage,” “elective cerclage,” “emergency cerclage,” and “pregnancy outcomes” were used to identify studies that reported pregnancy outcomes in women undergoing emergency and elective cerclages. The entire selection process was systematically documented using a PRISMA flow diagram.^7^ The protocol was registered at PROSPERO: CRD42024546566

Search strategy employed in PubMed is as follows:

(“Cervical Cerclage”[Mesh] OR cerclage[tiab] OR “cervical stitch”[tiab] OR “cervical suture”[tiab] OR cerclage*[tiab]) AND (“emergency cerclage”[tiab] OR “rescue cerclage”[tiab] OR “physical exam indicated cerclage”[tiab] OR “physical examination–indicated cerclage”[tiab] OR “ultrasound-indicated cerclage”[tiab] OR “history-indicated cerclage”[tiab] OR “prophylactic cerclage”[tiab] OR elective[tiab]) AND (“Pregnancy Outcome”[Mesh] OR “pregnancy outcome”[tiab] OR “preterm birth”[tiab] OR “preterm delivery”[tiab] OR “perinatal outcome”[tiab] OR “perinatal mortality”[tiab] OR “fetal survival”[tiab]) AND (“randomized controlled trial”[pt] OR random*[tiab] OR cohort[tiab] OR observational[tiab] OR prospective[tiab] OR retrospective[tiab])

### Inclusion Criteria:

Studies that explored the outcomes of pregnancies where cervical cerclage was performed, specifically differentiating between elective and emergency procedures, were included. Elective cerclages were typically history-guided, often based on previous obstetric history without immediate obstetric indications at the time of cerclage. Emergency cerclages were guided by physical examination findings or ultrasonographic evidence of cervical change, indicating an imminent risk to the ongoing pregnancy.

All types of observational studies, focusing on pregnant women who underwent either form of cerclage was considered. The primary comparison was between outcomes associated with elective and emergency cerclage. Primary outcomes included the rate of preterm birth before 37 weeks and before 28 weeks, gestational age at the time of cerclage placement, the incidence of premature rupture of membranes (PROM), vaginal delivery rates and neonatal birth weight.

### Study Selection:

For the selection of studies, the initial screening involved independently evaluating titles and abstracts by two reviewers to identify articles potentially relevant to our research focus. Subsequent to this screening, full-text articles deemed potentially suitable were retrieved and independently assessed for their inclusion in the study by the same reviewers. Any disagreements that arose during this phase were reconciled through mutual discussion until a consensus was reached.

### Data Collection Process:

In terms of data collection, we employed a meticulously designed standardized form to facilitate the extraction of data from the included studies. This Form was used by two independent reviewers to extract crucial information systematically. The data collected included detailed descriptions of the study settings, participant demographics, specific details about the cerclage intervention (elective or emergency), comparator characteristics and a comprehensive range of outcomes.

### Quality score assessment:

Potential biases in the included studies were evaluated using the Newcastle-Ottawa Scale (NOS) for observational studies.^8^ This scale comprises three critical domains: selection, comparability and outcome. Each study was systematically rated across these domains to identify the risk of bias as low (7-9 points), moderate (4-6 points), or high (< 4 points).

### Statistical analysis:

Data were analysed using a random-effects meta-analysis model employing the inverse variance method to accommodate the variability across studies. For categorical outcomes, frequency of events and sample sizes were recorded and the results were expressed as pooled odds ratios (OR) with 95% confidence intervals (CI). For continuous outcomes like gestational age at cerclage and neonatal birth weights, weighted mean differences (WMD) were calculated to reflect the average differences along with 95% CI.^9^ P-< 0.05 was considered statistically significant. Heterogeneity was quantitatively assessed using the I² statistic, with I² value >50% indicative of considerable heterogeneity.^9^

### Publication bias assessment:

Publication bias was assessed by funnel plots and Egger’s regression test. P< 0.05 in Egger’s test was taken as evidence of significant publication bias, suggesting an asymmetry in the distribution of studies that could influence the review’s conclusions. Publication bias assessment could not be done as less than 10 studies reported the outcome. All analyses were performed using the statistical software STATA 17.

### GRADE assessment:

We assessed the certainty of evidence for each key outcome using the GRADE approach. Two reviewers independently evaluated domains of risk of bias, inconsistency, indirectness, imprecision and publication bias, with discrepancies resolved by consensus. We rated evidence as high, moderate, low, or very low certainty.^9^

## RESULTS

**Fig.1:**
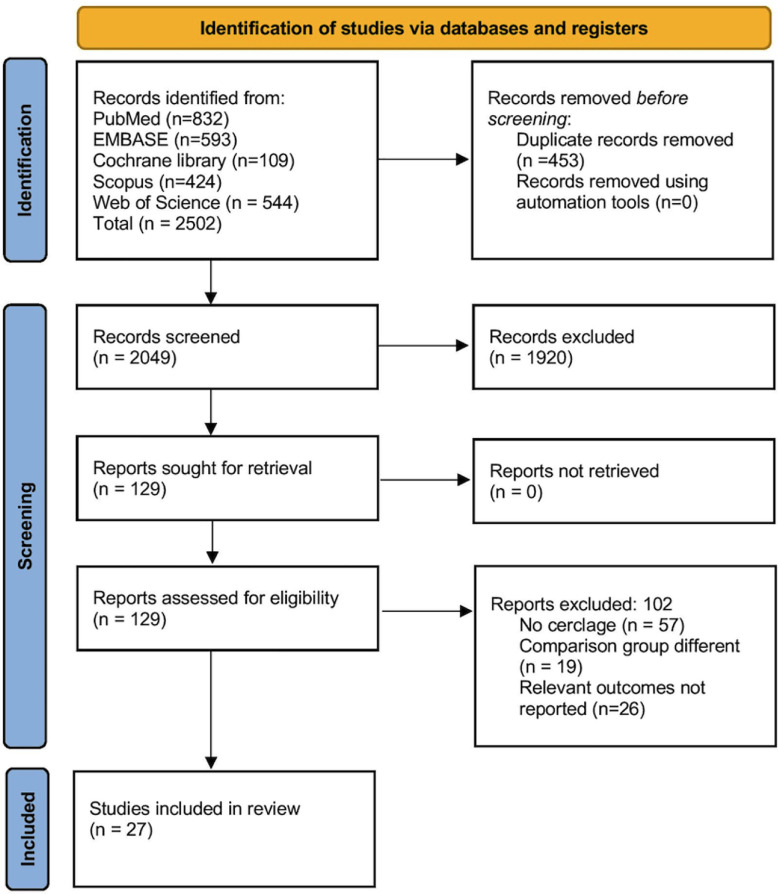
PRISMA flowchart.

An initial search across PubMed, EMBASE, Cochrane Library, Scopus and Web of Science databases identified 2,502 records. After removing 453 duplicates, titles and abstracts of 2,049 records were screened and 1,920 of them excluded. Full-text assessment was conducted for 129 articles, with 27 studies meeting our inclusion criteria. This process is visually represented in the accompanying PRISMA flow diagram. (Fig.1).^5^,^10^-^35^

### Characteristics of the included studies:

Table-I summarizes the results from 27 retrospective studies conducted across multiple countries, predominantly in Asia (India, China and Thailand). Total sample sizes ranged from 14 to 350 participants. Mean maternal ages ranged between 21 and around 35 years. Risk of bias assessments identified 11 studies with low, 10 studies with average and 6 studies with high risk of bias (Table-II).

**Table-I:** Characteristics of the included studies.

Author and year of publication	Location	Study design	Participant details	Sample size	Gestational age at delivery	Mean age in years	ROB
Nelson et al. 2009	USA	Retrospective study	Patients with singleton pregnancy who received a cervical cerclage placement which is either electively or urgently or emergently	Total :133 USG :26 PEG :18 HG :89	USG :34.2+5.9 PEG :29.3+7.2 HG :35.9+5.1	USG :24.5+5.7 PEG: 23.5+5.2 HG :25.7+5.1	Low
Kuruma et al 2022	Japan	Retrospective study	Women with singleton pregnancies who underwent cervical cerclage placement at Osaka Women’s and Children’s Hospital between January 2008 and December 2019.	Total :279 USG :96 PEG :145 HG :38	USG :38± 2.8 PEG: 34 ± 4 HG :38 ±2.75	USG :33 ± 4.1 PEG: 33± 4.3 HG: 34. ±5. 5	Moderate
Jafarzade et al 2024	Turkey	Retrospective study	Pregnant women with singleton pregnancies who underwent vaginal cervical cerclage with the McDonald technique between January 2017 and October 2022.	Total :247 PEG :142 HG :105	PEG: 30.8 HG: 34.6	PEG :30.5 HG :31.6	Moderate
Chen et al 2020	China	Retrospective study	Singleton Pregnant women who underwent cervical cerclage between January 2004 and October 2018	Total :326 USG :59 PEG :35 HG :232	USG :35.4 ± 2.3 PEG: 26.1± 1.0 HG :37.3± 0.9	USG :30.80 ± 4.40 PEG :31.66 ± 5.21 HG :30.36 ± 4.28	High
Khan et al 2012	UAE	Retrospective study	Pregnant women who had cervical cerclage performed in the period from January 2004 until December 2008 with mean age of 30 years.	Total :145 USG :16 PEG :17 HG :112	USG :35.7 ± 5.1 PEG: 32.4 ± 7.1 HG: 36.0 ± 5.1	USG: 29.12 ± 4.22 PEG :30.76 ± 5.39 HG :30.63 ± 5.21	Low
Gluck et al 2016	Israel	Retrospective study	Patients with a singleton pregnancy, who underwent cervical cerclage between January 2006 and December 2014 and delivered or underwent an abortion in a single tertiary medical centre	Total :201 USG :47 HG :154	USG :35.6±3 HG :36.1±3	USG :33.05±6.4 HG :32.6±6.1	Moderate
Golbasi et al 2022	Turkey	Retrospective study	Patients who were diagnosed with cervical insufficiency between January 2016 and December 2020	Total :73 USG :17 PEG :15 HG :41	USG :36.1±4.2 PEG :31.7±5.2 HG :34.9±5.3	USG :30.5±5.7 PEG :28.1±4.9 HG :29±6	Low
Kuon et al 2015	Germany	Retrospective study	Pregnant women identified with history of 1 preterm birth < 34 gestational weeks and/ or second trimester loss, cervical length < 25 mm before gestational week 24 during current pregnancy and cervical dilatation 2 cm and/ or bulging membranes	Total :100 USG :29 PEG :33 HG :38	USG :32± 4.25 PEG: 28 ±5.25 HG: 35± 5.25	USG: 32 ±3 PEG :33± 4 HG :33± 4.25	Low
Liddiard et al 2011	UK	Retrospective study	Patients who had a cervical cerclage inserted during pregnancy between 1985 and 2009.	Total :177 USG :25 PEG :9 HG :116	USG :31± 5.7 PEG :27.5± 4.0 HG :35± 4.1	USG :27.5± 5.0 PEG: 30.5 ±5.1 HG :31± 3.8	Moderate
Seyama et al 2022	Japan	Retrospective study	Multiparous women with a history of preterm birth were included.	Total :489 USG :56 PEG :45 HG :388	NR	USG :32.96 (4.74) PEG :32.47 (4.94) HG :32.74 (4.72)	Moderate
Mor et al 2023	Israel	Retrospective study	Women with a singleton pregnancy who underwent cervical cerclage between December 2008 and November 2021	Total :215 PEG :32 HG :183	PEG :35.8+4.7 HG :36.3+4.9	PEG :30.9±5 HG :32.4±5.5	Low
Simsek et al 2021	Turkey	Retrospective study	Singleton pregnancies in patients who underwent transvaginal cervical cerclage procedures over a 7-year period at a tertiary referral center.	Total: 75 PEG :27 HG :48	PEG :33.6±5.9 HG :35.6±4.5	PEG :31.7±4.4 HG :29.9±4.4	Moderate
Vasudeva et al 2018	Australia	Retrospective review	All singleton pregnant women who underwent an elective or emergency cervical cerclage between February 2014 and May 2017 at a single secondary obstetric centre.	Total :39 USG :13 HG :26	USG :34 HG :37.0	USG :30.6 HG :31.7	Low
Kumari et al 2023	India	Retrospective study	All pregnant women, both with singleton and twin pregnancies, who had cervical cerclage between June 2014 and May 2019	Total :129 USG/PEG :48 HG :81	USG/PEG:32.2±6.7 HG :34.2±7.0	USG/PEG:27.4±5.89 HG :29.06±4.62	High
Adekola et al 2022	USA	Retrospective study	Pregnant women who underwent transvaginal cerclage over a 4-year period were included	Total :72 USG :18 PEG :15 HG :39	USG: 34.99 ± 1.29 PEG: 34.38 ± 0.97 HG :35.92 ± 0.88	USG :31.28 ± 1.09 PEG :31.28 ± 1.09 HG :31.80 ± 0.85	Low
Wu et al 1996	Taiwan	Retrospective study	Patients undergone one or more cervical cerclage procedures between January 1982 to December 1994	Total :69 PEG :21 HG :48	PEG :28.3 ± 7.8 HG :31.0 ± 3.1	PEG :29.9 ± 4.8 HG :31.1± 4.8	Low
Cardosi et al 1998	Florida	Retrospective review	Patients who underwent cervical cerclage over a 7-year period from 1990-1996.	Total :40 USG :15 PEG :18 HG :7	USG :33 ±1.9 PEG :28 ± 1.5 HG :37 ±1.1	Not reported	Moderate
Guzman et al 1998	USA	Retrospective study	Women at risk for pregnancy loss and/or early spontaneous preterm birth are treated with an elective (planned) cervical cerclage.	Total :138 USG :57 HG :81	USG :37.0 ± 6 HG :37 ± 4.3	USG: 27 ± 6 HG :32 ±3.8	Moderate
Palaniappan et al 2002	UK	Retrospective study	Patients with singleton pregnancies and a history of one or more spontaneous losses between 16 and 33 weeks were included.	Total :90 USG :47 HG :43	USG :36.2 ± 6.4 HG :36.6 ± 4.5	NR	Moderate
Mullin et al 2023	UK	Retrospective study	Pregnant women who had a history of single spontaneous preterm birth or mid-trimester loss and also with a singleton.	Total :189 USG :123 HG :66	USG :39 +1 HG :28+3	USG :33.4 HG :34.0	Low
Chawanpaiboon et al 2010	Thailand	Retrospective study	Cervical insufficiency with history of recurrent pregnancy loss during 20-24 weeks of gestation between January 2006 and December 2008	Total :14 USG :8 HG :6	USG :28.7 ±3.4 HG :35.2 ±4.6	USG :34.0 ±1.1 HG :32.5 ±4.2	Moderate
Wang et al 2017	China	Retrospective study	Women with a viable singleton pregnancy, diagnosed with cervical insufficiency and underwent cervical cerclage between January 2010 and July 2015	Total :121 PEG :53 HG :68	PEG :33.50 ± 5.60 HG :34.79 ± 5.88	PEG :31.42 ± 4.35 HG :31.74 ± 4.85	High
Kurup et al 1999	Georgia	Retrospective study	Patients who underwent cervical cerclage placement between 1993-1997	Total :88 USG :15 PEG :35 HG :38	USG :33.07 ± 1.4 PEG :30.5 ± 0.9 HG :35.5 ± 0.8	USG :27.2 ± 4.5 PEG :26.2 ± 5.0 HG: 27.6 ± 4.7	High
Rodriguez 2020	New York	Retrospective study	Women with singleton pregnancies who underwent Shirodkar cerclage placement by a single maternal-fetal medicine practice between 2005 and 2019.	Total :350 USG :137 PEG :59 HG :154	NR	USG : 33.8 ± 5.7 PEG :35.2 ± 5.5 HG : 33.8 ± 5.3	Low
Frenken et al 2022	Netherlands	Retrospective study	All pregnant women who underwent cervical cerclage procedure between January 2000 to June 2020	Total :162 USG :16 PEG :17 HG :123	USG :29+6 PEG :38+5 HG :38+5	NR	Low
Gupta et al 2022	India	Retrospective study	Pregnant women who visited tertiary care centres were admitted with high risk of delivery.	Total :14 USG/PEG :4 HG :10	NR	USG/PEG :21- 30 years of age HG :21- 30 years of age	High
Liu et al.2018	China	Retrospective study	Pregnant women who underwent McDonald cervical cerclage in China between June 2014 and September 2016	Total 69 USG/PEG:30 HG: 39	USG/PEG:35.2 ± 5.5 weeks HG: 31.7 ± 6.5 weeks	USG/PEG:29.9 ± 3.4 HG: 30.3 ± 4.7	High

HG - History guided; PEG - Physical examination guided; USG - Ultrasonography guided

**Table-II:** Risk of bias assessment amongst included studies.

Author and year of publication	Selection	Comparability	Outcome	Overall score	ROB
Nelson et al. 2009	3 points	2 points	3 points	8 points	Low
Kuruma et al 2022	2 points	2 points	2 points	6 points	Moderate
Jafarzade et al 2024	2 points	2 points	1 point	5 points	Moderate
Chen et al 2020	1 point	0 points	1 point	2 points	High
Khan et al 2012	4 points	2 points	3 points	9 points	Low
Gluck et al 2016	1 point	2 points	2 points	5 points	Moderate
Golbasi et al 2022	3 points	2 points	3 points	8 points	Low
Kuon et al 2015	3 points	2 points	3 points	8 points	Low
Liddiard et al 2011	2 points	2 points	1 point	5 points	Moderate
Seyama et al 2022	2 points	2 points	1 point	5 points	Moderate
Mor et al 2023	2 points	2 points	3 points	7 points	Low
Simsek et al 2021	2 points	2 points	2 points	6 points	Moderate
Vasudeva et al 2018	3 points	2 points	3 points	8 points	Low
Kumari et al 2023	2 points	0 points	1 point	3 points	High
Adekola et al 2022	3 points	2 points	3 points	8 points	Low
Wu et al 1996	2 points	2 points	3 points	7 points	Low
Cardosi et al 1998	3 points	1 point	2 points	6 points	Moderate
Guzman et al 1998	2 points	2 points	2 points	6 points	Moderate
Palaniappan et al 2002	1 point	1 point	3 points	5 points	Moderate
Mullin et al 2023	3 points	2 points	3 points	8 points	Low
Chawanpaiboon et al 2010	2 points	2 points	2 points	6 points	Moderate
Wang et al 2017	1 point	0 points	1 point	2 points	High
Kurup et al 1999	3 points	2 points	3 points	8 points	High
Rodriguez 2020	3 points	2 points	3 points	7 points	Low
Frenken et al 2022	2 points	2 points	3 points	7 points	Low
Gupta et al 2022	1 point	1 point	1 point	3 points	High
Liu et al.2018	1 point	0 points	2 points	3 points	High

### Gestational age at cerclage:

**Fig.2:**
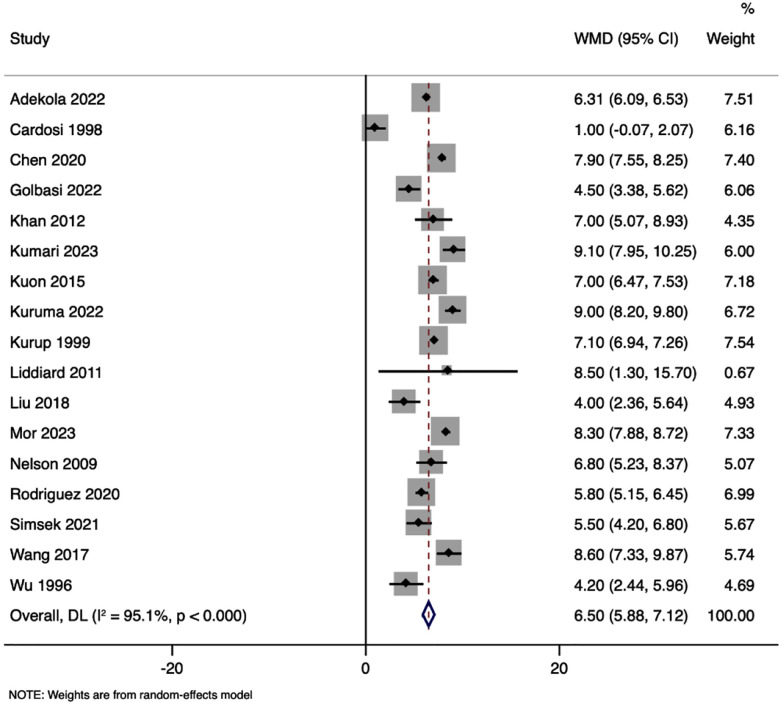
Forest plot showing the differences in gestational age at cerclage between physical examination guided and history guided cervical cerclage.

There was a significant overall effect with a WMD of 6.500 weeks (95% CI: 5.885 to 7.116) in the physical examination-guided group compared to the history-guided elective approach group, with substantial heterogeneity (I² = 95.1%, p < 0.001) Fig.2.

The analysis revealed a significant overall effect, with a WMD of 5.698 weeks (95% CI: 4.951 to 6.445; p<0.001) in the ultrasound-guided emergency group compared to the history-guided elective group. There was a marked heterogeneity (I² = 94.9%, p < 0.001). The funnel plot analysis and Egger’s test (p<0.001) indicated the presence of publication bias.

### Neonatal birth weight:

**Fig.3:**
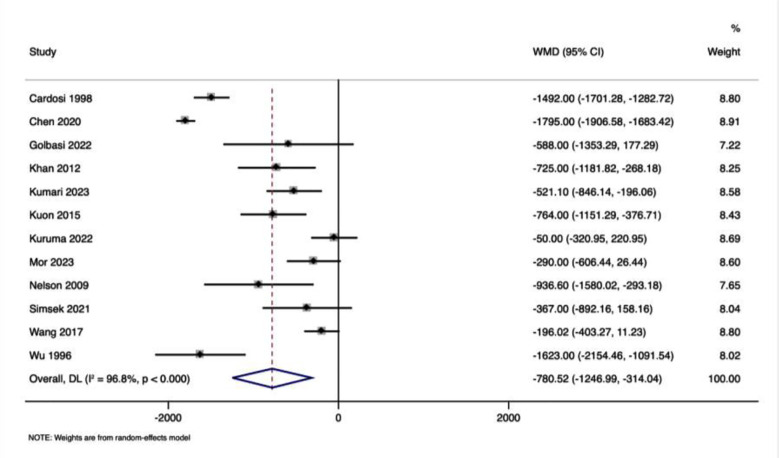
Forest plot showing the differences in neonatal birth weight between physical examination guided and history guided cervical cerclage.

A pooled WMD of -780.52 (-1246.99, -314.04) indicated a significant reduction in neonatal birth weight in offspring of women who underwent physical examination-guided emergency cerclage compared to history-guided elective cerclage (Fig.3), with considerable heterogeneity (I² = 96.8%, p < 0.001). The funnel plot was asymmetrical, indicating the presence of publication bias, further confirmed by the significant Egger’s test (p<0.001). The analysis showed pooled WMD of -281.853 grams (95% CI: -454.910 to -108.796; p=0.001; I²=65.2% Cochran’s Q p = 0.003), indicating that neonates from pregnancies managed by ultrasound-guided emergency cerclage tended to have lower birth weights compared to the elective cerclage group.

### PROM:

**Fig.4:**
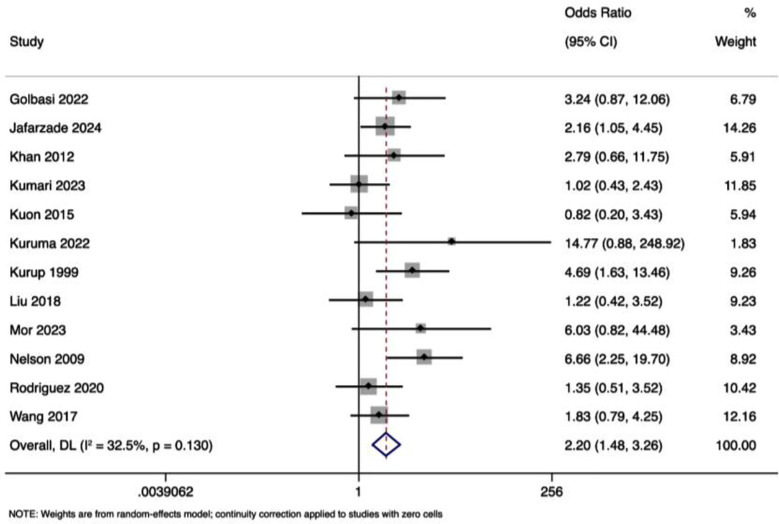
Forest plot showing the differences in premature rupture of membranes between physical examination guided and history guided cervical cerclage.

Pooled OR was 2.197 (95% CI: 1.479 to 3.264), indicating a significantly increased risk of PROM associated with physical examination-guided emergency cerclage compared to elective cerclage (z = 3.901, p < 0.001) (Fig.4). The heterogeneity among the studies was moderate (I² =32.5%; Cochran’s Q = 16.30, p = 0.130). The funnel plot was symmetrical, further confirmed by a non-significant Egger’s test (p=0.18). Pooled OR of 1.445 (95%CI: 0.911 to 2.292) indicated that the risk of PROM was similar in the two cerclage approaches (z = 1.563, p = 0.118). The heterogeneity among the included studies was very low, (I² = 1.9%) and p = 0.418 on Cochran’s Q statistic.

### Vaginal delivery rate:

**Fig.5:**
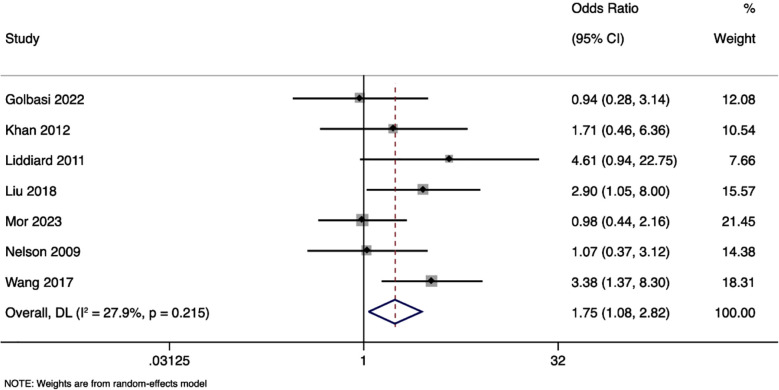
Forest plot showing the differences in vaginal delivery rates between physical examination guided and history guided cervical cerclage.

The analysis showed a pooled OR of 1.750 (95%CI: 1.085 to 2.824), indicating that the emergency cerclage approach was associated with a significantly higher likelihood of vaginal delivery (z = 2.294, p = 0.022) (Fig.5). Heterogeneity across the included studies was low (I²=27.9%; Cochran’s Q p = 0.215). Both approaches (ultrasound-guided emergency *and* history-guided elective cerclage) were associated with a comparable rate of vaginal delivery, with the pooled OR of 0.768 (95%CI: 0.523 to 1.128; z = -1.347, p = 0.178), with no heterogeneity (I² = 0.0%, p = 0.625).

### Preterm birth at 37 weeks:

Pooled OR for preterm birth before 37 weeks in women managed by physical examination-guided emergency cerclage compared to history-guided elective cerclage was 2.272 (95%CI: 1.515 to 3.406), which indicated a significantly higher risk of preterm birth before 37 weeks that was associated with emergency cerclage (z = 3.971, p < 0.001). Heterogeneity was moderate (I²=48.2%, Cochran’s Q = 25.08, p = 0.023). Funnel plot was symmetrical as further confirmed by non-significant Egger’s test (p=0.25). The pooled OR of 2.135 (95%CI: 1.480 to 3.081) was indicative of the significantly higher risk of preterm birth before 37 weeks in women who underwent ultrasound-guided emergency cerclage (z = 4.056, p < 0.001). The heterogeneity among the studies was moderate to high (I² of 54.4%, Cochran’s Q = 28.53, p = 0.008), with asymmetrical funnel plots and significant Egger’s test (p=0.03).

### Preterm birth at 28 weeks:

Pooled OR of 3.848 (95%CI: 1.893 to 7.818; z = 3.725, p < 0.001) was found for preterm birth before 28 weeks in women with physical examination-guided emergency cerclage compared to history-guided elective cerclage, indicating significantly higher risk of preterm birth before 28 weeks in women after emergency cerclage. Heterogeneity was substantial (I² of 61.5; Cochran’s Q = 23.37, p = 0.005). The funnel plot was symmetrical and Egger’s test was insignificant (p=0.96).

The pooled OR of 2.361 (95%CI: 1.056 to 5.282) indicated that ultrasound-guided emergency cerclage was associated with a significantly higher risk of very early preterm birth (z = 2.092, p = 0.036). The heterogeneity was substantial (I²=62.0%, Cochran’s Q = 21.04, p = 0.007).

### Certainty of evidence:

The GRADE assessment indicated moderate-certainty evidence for the pooled reduction in preterm delivery with elective cerclage (downgraded one level for inconsistency) and low-certainty evidence for perinatal mortality comparisons (downgraded two levels for imprecision and risk of bias).

## DISCUSSION

Our meta-analysis provided several critical insights into the outcomes associated with physical examination-guided emergency cerclage and ultrasound-guided emergency cerclage, compared to history-guided elective cerclage. Our study found that emergency cerclage placement occurred significantly later in gestation compared to the elective placement (a WMD of 6.500 weeks and 5.698 weeks for examination-guided and ultrasound-guided emergency cerclages, respectively). Physical examination-guided emergency cerclage was associated with a significantly lower neonatal birth weight in offspring compared to the elective cerclage, with a WMD of -780.52 grams. This reduction in birth weight was also noted in women who underwent ultrasound-guided cerclage, albeit to a lesser extent (WMD of -281.853 grams). Our findings align with earlier research that suggests that emergency cerclage is often a reactionary measure that is taken when risk factors for preterm birth, such as cervical shortening or funnelling, are identified later in pregnancy.^6^,^12^-^15^,^23^-^27^,^36^

There is a substantial variability in the previous reports on the success rates of elective versus emergency cerclages in preventing preterm birth and improving neonatal outcomes.^6^,^22^-^30^,^36^ Our study showed that emergency cerclage, especially when guided by physical examination, is associated with higher rates of PROM and preterm birth. These results are consistent with some prior studies but contrast with other reports that detected similar outcomes between emergency and elective cerclage when adjusted for confounders like gestational age at cerclage and baseline cervical length.^11^-^30^,^36^ These studies corroborate the notion that later intervention may correlate with less optimal pregnancy outcomes, such as reduced birth weights and increased rates of PROM.

This study also showed that physical examination-guided emergency cerclage is associated with an increased likelihood of vaginal delivery. This is a novel finding that has not been widely reported and may reflect a bias towards non-operative vaginal delivery in cases where cerclage was deemed necessary due to acute changes in cervical status.

Both emergency cerclage methods in our study were linked to a significantly higher risk of preterm birth before 37 and 28 weeks. This risk was particularly pronounced in the physical examination-guided group at 28 weeks (OR of 3.848). In terms of preterm birth at both 37 and 28 weeks, our findings further underscore the elevated risk associated with emergency cerclage. Our results support the hypothesis that conditions necessitating emergency cerclage are inherently associated with higher obstetric risk. This is consistent with existing literature which often reports adverse outcomes associated with emergent procedures.^6^,^36^

Our GRADE assessment revealed that risk of bias was a key driver in downgrading the certainty of evidence for several outcomes. Many of the included observational studies were rated at moderate to high risk particularly due to confounding (limited adjustment for key prognostic factors), selection bias (non-randomized enrolment) and incomplete outcome data which led us to downgrade the certainty of preterm birth estimates by one level and perinatal mortality by two levels. This pattern underscores that, while pooled effect estimates suggest benefits of elective over emergency cerclage, the underlying study limitations warrant cautious interpretation and highlight the need for more rigorous prospective trials.

The mechanisms underlying the differences in outcomes between emergency and elective cerclage can be attributed to several factors. The gestational age at which the cerclage is placed is crucial. Elective cerclage is typically performed earlier in the pregnancy before significant cervical changes have occurred.^3^ In contrast, emergency cerclage is often a response to observed cervical insufficiency, which may already be at an advanced stage.^4^ This delay can increase the risk of inflammation, infection and further weakening of the cervical tissue, potentially explaining the higher rates of PROM and preterm birth observed in these groups.^6^ Moreover, patients who require emergency cerclage are likely to have different baseline obstetric risk profiles compared to those receiving elective cerclage. It is plausible, therefore, that underlying conditions, such as a history of cervical insufficiency or previous preterm births, may contribute to the higher observed rates of adverse outcomes.^6^,^36^

Additionally, emergency cerclage procedures might induce more physiological stress on both the mother and the fetus due to the urgency and the conditions necessitating the intervention. This stress could contribute to higher rates of complications such as PROM and adverse neonatal outcomes. The technique and circumstances of cerclage placement might also play a role due to the urgent nature and differing practitioner experiences, potentially leading to less optimal outcomes.

### Strengths:

To our knowledge, this is the first meta-analysis to directly contrast emergency and elective cerclage approaches. Previous reviews have characterized benefits of individual cerclage indications but lacked a head-to-head quantitative comparison.^3^ By integrating data across both strategies, our findings deliver novel, evidence-based guidance on selecting the most effective timing for cervical cerclage. We employed robust meta-analytical techniques, including random-effects models and continuity corrections for zero cells, to provide a thorough analysis of the available data. The use of standardized protocols for data extraction, assessment of study quality and statistical analysis ensured a systematic and unbiased approach to synthesizing the existing literature.

### Limitations:

Although the review used random-effects models to account for variability among studies, considerable heterogeneity was present in some outcomes. This suggests that the results should be interpreted with caution, considering the potential differences in study populations, methodologies and definitions of outcomes. The presence of publication bias, as indicated by asymmetrical funnel plots and significant Egger’s test results for some comparisons, suggests that the findings may be influenced by selective publication of studies with positive results. All the included studies were non-randomized retrospective or prospective designs, limiting internal validity due to potential selection bias and unmeasured confounding. Key prognostic factors such as gestational age at cerclage placement, cervical dilation and membrane status were variably reported and rarely adjusted for in analyses. This heterogeneity in study design and lack of randomization constrains causal inference and underscore the need for prospective, controlled trials with rigorous confounder control.

The findings of this review have several important implications for clinical practice. Clinicians should consider the timing and indications for cerclage more carefully, especially distinguishing between patients who may benefit from elective *versus* emergency cerclage. Risk factors for adverse outcomes should be thoroughly assessed to tailor the intervention more effectively to the patient’s specific needs. Increased monitoring and proactive management may be necessary for patients undergoing emergency cerclage, given the higher risk of PROM and preterm birth. This could include more frequent ultrasound examinations and possibly the administration of agents to enhance fetal lung maturity or prevent infection.

Patients who are candidates for emergency cerclage should be counselled about the increased risks associated with the procedure compared to elective cerclage. This counselling should include discussions of potential outcomes and steps to mitigate risks. Efforts should be made to standardize the technique and conditions under which emergency cerclages are performed to reduce variability in outcomes and ensure the best possible results for patients. Emergency cerclage patients often present with advanced cervical dilation or membrane prolapse; conditions linked to inherently higher preterm birth risk introducing confounding by indication. Future studies should adjust for these baseline differences (e.g., via propensity matching) to better isolate the effect of cerclage timing.

In practice, obstetricians must balance the clear benefits of elective cerclage against the risks of an unnecessary procedure. For women with well-established risk factors such as a history of spontaneous preterm birth or ultrasound-detected cervical shortening (<25 mm) before 14 weeks’ gestation elective cerclage should be offered proactively, as it maximizes term delivery rates and minimizes preterm birth risk. Conversely, when cervical changes are first noted later in pregnancy or in the context of membrane prolapse, emergency cerclage remains a valuable salvage intervention despite its comparatively lower efficacy. Decision-making should therefore integrate individual patient history, timing of cervical insufficiency signals and patient preferences, ensuring that elective cerclage is prioritized in high-risk scenarios while reserving emergency cerclage for true rescue situations.

### Recommendations for Future Research:

Future research could address several gaps identified in this review. Prospective longitudinal studies are needed to better understand the long-term outcomes of different cerclage techniques and clarify the causal mechanisms behind the observed associations. Where ethical and feasible, randomized controlled trials comparing emergency and elective cerclage could provide higher-quality evidence to guide clinical decisions. Further studies should consider subgroup analyses based on factors like the severity of cervical insufficiency, the presence of fetal fibronectin, or biomarkers of inflammation to refine the selection criteria for cerclage.

Research into new technologies or improvements in ultrasound imaging^37^ that could aid in the earlier detection of cervical changes might help in deciding the optimal timing for cerclage placement. Including diverse populations from different geographic and socioeconomic backgrounds in future studies would enhance the generalizability of the findings and ensure that recommendations are applicable globally.

## CONCLUSION

This meta-analysis shows that elective cerclage was associated with a higher rate of term delivery and a lower risk of preterm birth (<34 weeks) compared with emergency cerclage, while perinatal mortality did not differ significantly between the two approaches. These findings support the preferential use of elective cerclage when risk factors are identified antenatally.

### Authors’ contributions:

**YZ:** Study design and manuscript writing. Manuscript revision and validation, is responsible for the integrity of the study.

**YZ and TT:** Were involved in data collection, data analysis and interpretation. Critical Review.

All authors have read and approved the final manuscript.
